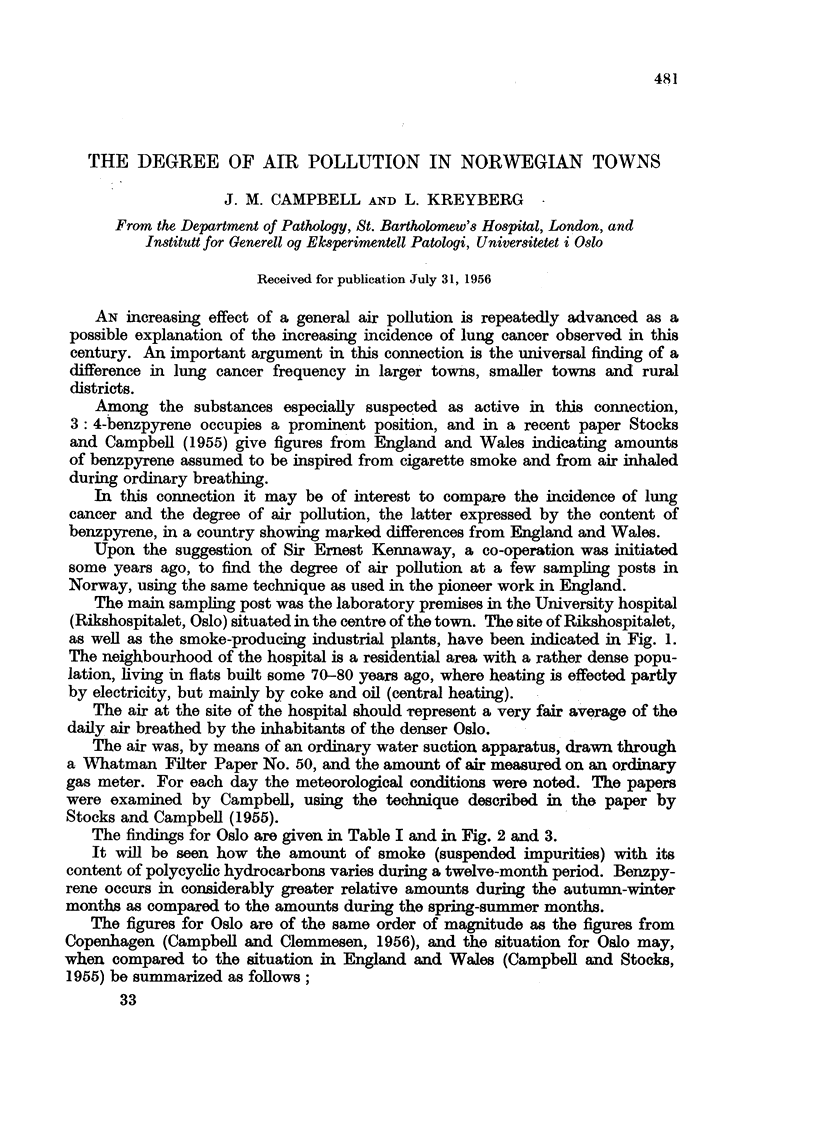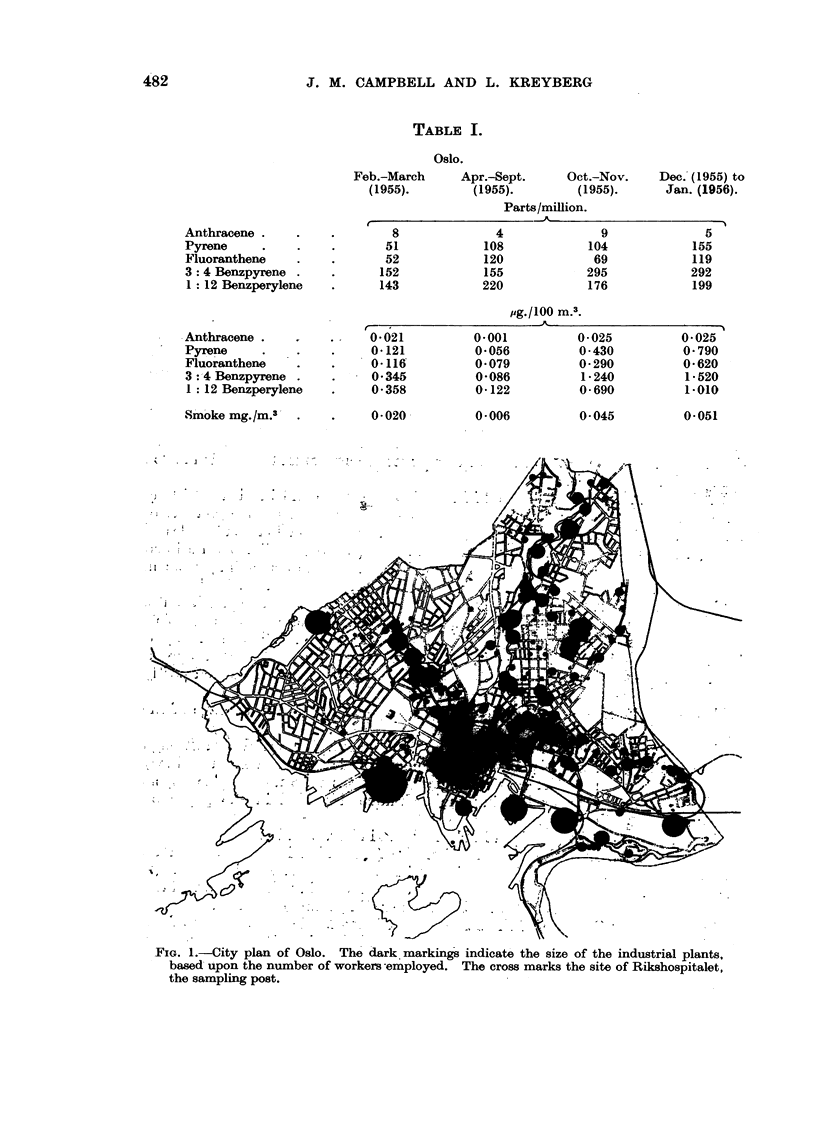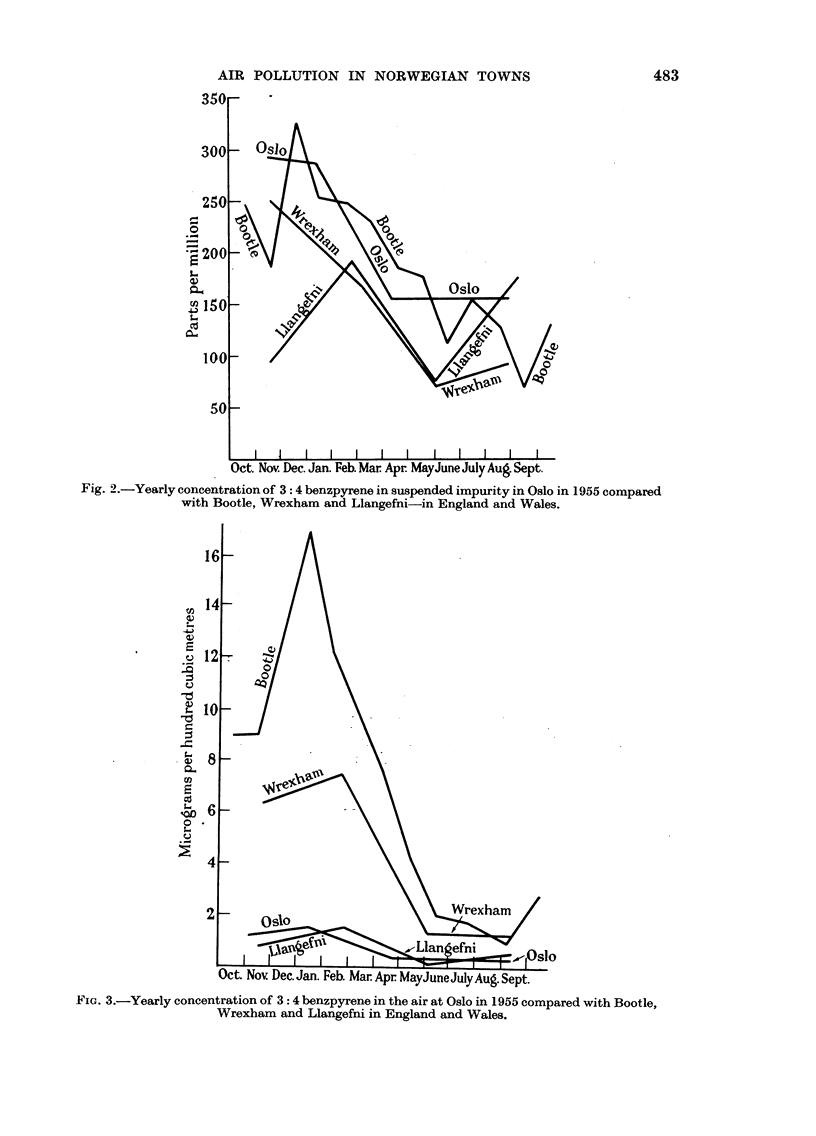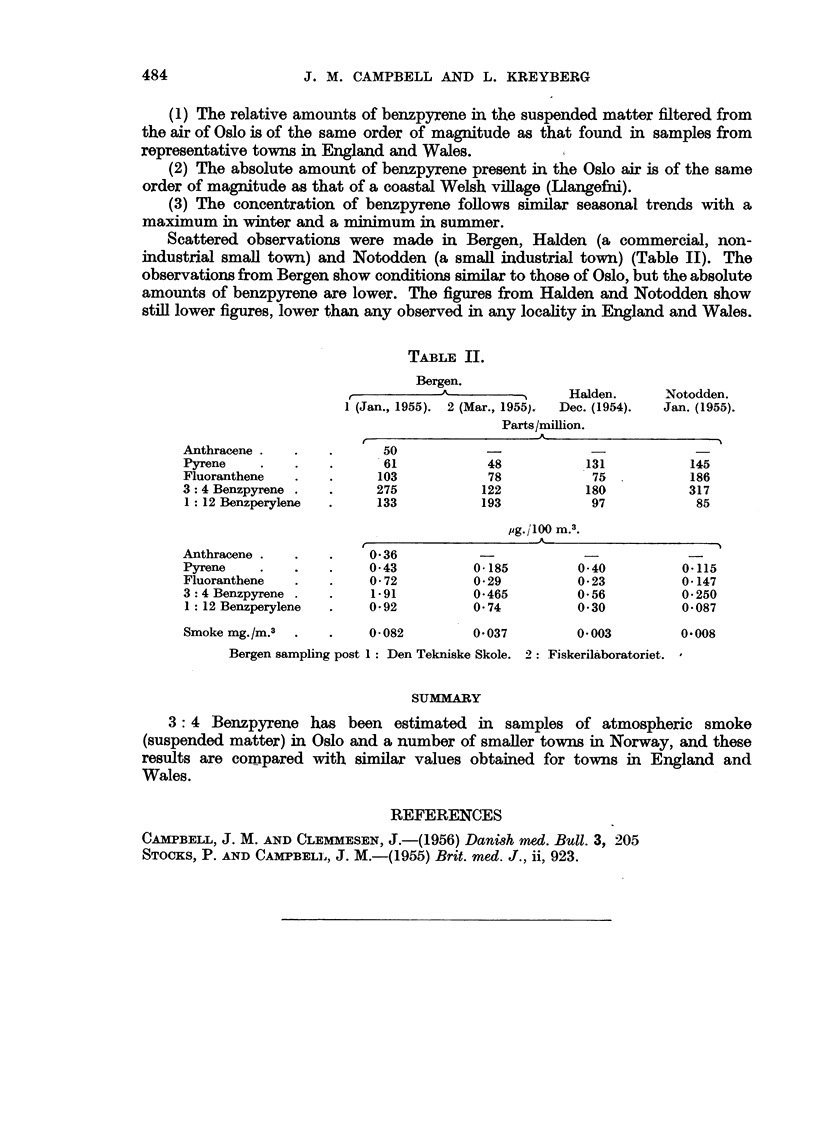# The Degree of Air Pollution in Norwegian Towns

**DOI:** 10.1038/bjc.1956.54

**Published:** 1956-09

**Authors:** J. M. Campbell, L. Kreyberg


### THE DEGREE OF AIR POLLUTION IN NORWEGIAN TOWNS

J. M. CAMPBELL AND L. KREYBERG

*From the Department of Pathology, St. Bartholomew's Hospital, London, and Institutt for Generell og Eksperimentell Patologi, Universitetet i Oslo*

Received for publication July 31, 1956

An increasing effect of a general air pollution is repeatedly advanced as a possible explanation of the increasing incidence of lung cancer observed in this century. An important argument in this connection is the universal finding of a difference in lung cancer frequency in larger towns, smaller towns and rural districts.

Among the substances especially suspected as active in this connection, 3 : 4-benzpyrene occupies a prominent position, and in a recent paper Stocks and Campbell (1955) give figures from England and Wales indicating amounts of benzpyrene assumed to be inspired from cigarette smoke and from air inhaled during ordinary breathing.

In this connection it may be of interest to compare the incidence of lung cancer and the degree of air pollution, the latter expressed by the content of benzpyrene, in a country showing marked differences from England and Wales.

Upon the suggestion of Sir Ernest Kennaway, a co-operation was initiated some years ago, to find the degree of air pollution at a few sampling posts in Norway, using the same technique as used in the pioneer work in England.

The main sampling post was the laboratory premises in the University hospital (Rikshospitalet, Oslo) situated in the centre of the town. The site of Rikshospitalet, as well as the smoke-producing industrial plants, have been indicated in Fig. 1. The neighbourhood of the hospital is a residential area with a rather dense population, living in flats built some 70–80 years ago, where heating is effected partly by electricity, but mainly by coke and oil (central heating).

The air at the site of the hospital should represent a very fair average of the daily air breathed by the inhabitants of the denser Oslo.

The air was, by means of an ordinary water suction apparatus, drawn through a Whatman Filter Paper No. 50, and the amount of air measured on an ordinary gas meter. For each day the meteorological conditions were noted. The papers were examined by Campbell, using the technique described in the paper by Stocks and Campbell (1955).

The findings for Oslo are given in Table I and in Fig. 2 and 3.

It will be seen how the amount of smoke (suspended impurities) with its content of polycyclic hydrocarbons varies during a twelve-month period. Benzpyrene occurs in considerably greater relative amounts during the autumn-winter months as compared to the amounts during the spring-summer months.

The figures for Oslo are of the same order of magnitude as the figures from Copenhagen (Campbell and Clemmesen, 1956), and the situation for Oslo may, when compared to the situation in England and Wales (Campbell and Stocks, 1955) be summarized as follows ;

TABLE I.

Oslo.

| | Feb.–March (1955). | Apr.–Sept. (1955). | Oct.–Nov. (1955). | Dec. (1955) to Jan. (1956). |
|---|---|---|---|---|
| | Parts/million. | | | |
| Anthracene | 8 | 4 | 9 | 5 |
| Pyrene | 51 | 108 | 104 | 155 |
| Fluoranthene | 52 | 120 | 69 | 119 |
| 3 : 4 Benzpyrene | 152 | 155 | 295 | 292 |
| 1 : 12 Benzperylene | 143 | 220 | 176 | 199 |
| | $\mu$g./100 m.$^3$. | | | |
| Anthracene | 0·021 | 0·001 | 0·025 | 0·025 |
| Pyrene | 0·121 | 0·056 | 0·430 | 0·790 |
| Fluoranthene | 0·116 | 0·079 | 0·290 | 0·620 |
| 3 : 4 Benzpyrene | 0·345 | 0·086 | 1·240 | 1·520 |
| 1 : 12 Benzperylene | 0·358 | 0·122 | 0·690 | 1·010 |
| Smoke mg./m.$^3$ | 0·020 | 0·006 | 0·045 | 0·051 |

FIG. 1.—City plan of Oslo. The dark markings indicate the size of the industrial plants, based upon the number of workers employed. The cross marks the site of Rikshospitalet, the sampling post.

Fig. 2.—Yearly concentration of 3 : 4 benzpyrene in suspended impurity in Oslo in 1955 compared with Bootle, Wrexham and Llangefni—in England and Wales.

Fig. 3.—Yearly concentration of 3 : 4 benzpyrene in the air at Oslo in 1955 compared with Bootle, Wrexham and Llangefni in England and Wales.

(1) The relative amounts of benzpyrene in the suspended matter filtered from the air of Oslo is of the same order of magnitude as that found in samples from representative towns in England and Wales.

(2) The absolute amount of benzpyrene present in the Oslo air is of the same order of magnitude as that of a coastal Welsh village (Llangefni).

(3) The concentration of benzpyrene follows similar seasonal trends with a maximum in winter and a minimum in summer.

Scattered observations were made in Bergen, Halden (a commercial, non-industrial small town) and Notodden (a small industrial town) (Table II). The observations from Bergen show conditions similar to those of Oslo, but the absolute amounts of benzpyrene are lower. The figures from Halden and Notodden show still lower figures, lower than any observed in any locality in England and Wales.

TABLE II.

| | Bergen. 1 (Jan., 1955). | Bergen. 2 (Mar., 1955). | Halden. Dec. (1954). | Notodden. Jan. (1955). |
|---|---|---|---|---|
| | Parts/million. | | | |
| Anthracene | 50 | — | — | — |
| Pyrene | 61 | 48 | 131 | 145 |
| Fluoranthene | 103 | 78 | 75 | 186 |
| 3 : 4 Benzpyrene | 275 | 122 | 180 | 317 |
| 1 : 12 Benzperylene | 133 | 193 | 97 | 85 |
| | $\mu$g./100 m.$^3$. | | | |
| Anthracene | 0·36 | — | — | — |
| Pyrene | 0·43 | 0·185 | 0·40 | 0·115 |
| Fluoranthene | 0·72 | 0·29 | 0·23 | 0·147 |
| 3 : 4 Benzpyrene | 1·91 | 0·465 | 0·56 | 0·250 |
| 1 : 12 Benzperylene | 0·92 | 0·74 | 0·30 | 0·087 |
| Smoke mg./m.$^3$ | 0·082 | 0·037 | 0·003 | 0·008 |

Bergen sampling post 1 : Den Tekniske Skole. 2 : Fiskerilåboratoriet.

### SUMMARY

3 : 4 Benzpyrene has been estimated in samples of atmospheric smoke (suspended matter) in Oslo and a number of smaller towns in Norway, and these results are compared with similar values obtained for towns in England and Wales.

### REFERENCES

CAMPBELL, J. M. AND CLEMMESEN, J.—(1956) *Danish med. Bull.* **3,** 205
STOCKS, P. AND CAMPBELL, J. M.—(1955) *Brit. med. J.*, ii, 923.